# Exogenous *Aspergillus aculeatus* Enhances Drought and Heat Tolerance of Perennial Ryegrass

**DOI:** 10.3389/fmicb.2021.593722

**Published:** 2021-02-19

**Authors:** Xiaoning Li, Chuncheng Zhao, Ting Zhang, Guangyang Wang, Erick Amombo, Yan Xie, Jinmin Fu

**Affiliations:** ^1^Coastal Salinity Tolerant Grass Engineering and Technology Research Center, Ludong University, Yantai, China; ^2^CAS Key Laboratory of Plant Germplasm Enhancement and Specialty Agriculture, Wuhan Botanical Garden, Chinese Academy of Sciences, Wuhan City, China

**Keywords:** perennial ryegrass, *Aspergillus aculeatus*, response mechanisms, drought stress, heat stress

## Abstract

Perennial ryegrass (*Lolium perenne*) is a cool-season grass whose growth and development are limited by drought and high temperature. *Aspergillus aculeatus* has been reported to promote plant growth and counteract the adverse effects of abiotic stresses. The objective of this study was to assess *A. aculeatus*-induced response mechanisms to drought and heat resistance in perennial ryegrass. We evaluated the physiological and biochemical markers of drought and heat stress based on the hormone homeostasis, photosynthesis, antioxidant enzymes activity, lipid peroxidation, and genes expression level. We found out that under drought and heat stress, *A. aculeatus*-inoculated leaves exhibited higher abscisic acid (ABA) and lower salicylic acid (SA) contents than non-inoculated regimes. In addition, under drought and heat stress, the fungus enhanced the photosynthetic performance, decreased the antioxidase activities, and mitigated membrane lipid peroxidation compared to non-inoculated regime. Furthermore, under drought stress, *A. aculeatus* induced a dramatic upregulation of *sHSP17.8* and *DREB1A* and a downregulation of *POD47*, *Cu/ZnSOD*, and *FeSOD* genes. In addition, under heat stress, *A. aculeatus*-inoculated plants exhibited a higher expression level of *HSP26.7a*, *sHSP17.8*, and *DREB1A* while a lower expression level of *POD47* and *FeSOD* than non-inoculated ones. Our results provide an evidence of the protective role of *A. aculeatus* in perennial ryegrass response to drought and heat stresses.

## Introduction

Plant growth and propagation are influenced by abiotic stressors such as high or low temperatures, heavy metal, or drought (Schramm et al., 2006; Qin et al., 2010). Heat stress is often accompanied by drought stress (Tester and Bacic, 2005). Thus, drought and heat can be primary limiting factors of plant growth, development and productivity, for cool-season turfgrass especially during summer (Jiang and Huang, 2001).

Studies have shown that drought and heat stresses often trigger pernicious effects on the photosynthetic apparatus (Ramachandra et al., 2004; Rennenberg et al., 2006). For instance, collectively, they may lead to distinctive loss of pigments and disorganize thylakoid membranes (Ladjal et al., 2000; Chen et al., 2013). In plants, photosynthesis, and especially the photochemistry of Photosystem II (PSII) has been confirmed to be the most heat-sensitive process (Berry and Björkman, 1980; Bilger et al., 1987; Al-Khatib and Paulsen, 1999). In addition, many studies have suggested that PSII is one of the primary sites of damage induced by a variety of stress factors, while PSII reaction center D1 proteins are the main targets of heat stress (Berry and Björkman, 1980; Niyogi, 1999; Yamamoto, 2001). After the D1-D2 heterodimer forms the PSII reaction center, it can bind all the fundamental cofactors for electron transfer (Yamamoto et al., 2008; Chen et al., 2013). Thus, once the D1-D2 proteins are damaged by heat stress, the photo-induced electron transfer from water to the plastoquinone is disrupted, and thereby affecting the photochemical efficiency of PSII (Chen et al., 2013).

Drought or/and heat stress can offset the balance between the production and utilization of photosynthetic electrons, and then eliciting oxidative stress by generating reactive oxygen species (ROS) such as hydroxyl (OH^−^), hydrogen peroxide (H_2_O_2_), superoxide radicals (O_2_^−^), and singlet oxygen (^1^O_2_;) (Feller, 2007; Harsh et al., 2016). Many studies have demonstrated that the overproduction of ROS result in enzyme inactivation and membrane damage (Apel and Hirt, 2004; Scandalios, 2005). To counteract the adverse effects of excessive ROS, plants have evolved a complex yet efficient ROS-scavenging system (Mittler, 2002; Harsh et al., 2016). The antioxidant enzymes consist of (but not limited to) peroxidase (POD; EC 1.11.1.7), superoxide dismutase (SOD; CE 1.15.1.1), and peroxidase (CAT; EC 1.11.1.6). Among the antioxidant enzymes, SOD is the first line in the active oxygen scavenging system and can specifically catalyze O_2_^−^ to H_2_O_2_ and O_2_, thus completing the removal of O_2_^−^; while the produced H_2_O_2_ is scavenged by POD and CAT (Harsh et al., 2016). Generally, these antioxidant enzymes possess multiple isoenzymes that act cooperatively in mitigating tissue injury and protecting cellular organelles (Mittler, 2002).

The synthesis and accumulation of compatible solutes, such as amino acid, proline, and soluble sugar, are often regarded as an adaptive strategy of plants to withstand the injury of drought and heat stresses (Harsh et al., 2016; Zegaoui et al., 2017). To cope with the osmotic stress, the compatible solutes are accumulated in plant cells to increase the osmotic pressure and prevent water loss (Ramachandra et al., 2004). A previous study in maize reported that an increase in the total soluble protein content decreased negative effects of drought stress (Mohammadkhani and Heidari, 2014). In addition, other investigators highlighted that plants responded to heat stress by producing and accumulating compatible solutes (e.g., carbohydrates, proline, and glycine betaine; Krasensky and Jonak, 2012; Hasanuzzaman et al., 2013). Thus, the production of compatible solutes is an effective approach of plants to enhance stress tolerance.

To counter the adverse influences of drought and heat stress, various defense genes such as ROS-scavenging enzymes and heat shock proteins (HSPs) are induced (Qin et al., 2010; Hu et al., 2011). Previous studies reported that HSPs are involved in plant thermotolerance (Li and Werb, 1982). Earlier studies demonstrated that *DREB2A* not only regulates *HSFA3* gene expression but also conferred heat and drought stress stresses tolerance to plants (Schramm et al., 2006; Qin et al., 2010). Concurrent with the physiological and molecular changes, plants growth and stress tolerance also benefit from mutually symbiotic fungi and bacteria (Baltruschat et al., 2008; Parniske, 2008). Many studies have characterized the drought-related genes, i.e., *CBL1*, *DREB2A*, and *RD29A*, which were induced by *Piriformospora indica* under drought stress (Chao et al., 2010; Xu et al., 2017). In agreement with those findings, it was reported that *P. indica* could confer drought tolerance to plants through altering the expression level of a series of stress-associated genes (Sherameti et al., 2008). Therefore, symbiotic microbe-induced plant protection strategy from the negative influences of drought and heat stresses is worthy of attention.

*Aspergillus aculeatus* was screened and isolated from the cadmium-contaminated soil (Xie et al., 2014). It has been reported that *A. aculeatus* had the capacity to dissolve insoluble phosphorus in nature, hence promoting its uptake, transport, and utilization (Narsian and Patel, 2000). Our previous studies testified that the *A. aculeatus* could promote photosynthesis and growth rate of plants with the production of indole-3-acetic acid (IAA) and siderophores ultimately enhancing tolerance to salt and Cd stresses. Therefore, the salt or/and Cd-induced inhibition of plants metabolic activity seemed to be mitigated by the *A. aculeatus* (Li et al., 2017; Xie et al., 2017a,b). Perennial ryegrass (*Lolium perenne* L.) is an extensively used forage grass due to its high nutritive values and herbage yield (Wilkins, 1991). In addition, it is widely used as a turfgrass in the park, roadsides, athletic fields, and other places due to its fast establishment and superior tillering capability (Hannaway et al., 1999). As a cool-season grass, its growth, reproduction, and development are limited by drought and high temperature stresses (Iwayainoue et al., 2004; Ren et al., 2004). However, the role of *A. aculeatus* in drought and heat tolerance of perennial ryegrass remains unclear.

Thus, to expound on the physiological responses of *A. aculeatus*-regulated perennial ryegrass to drought and heat stresses, important indicators such as the membrane stabilization, antioxidant enzymes activities, photosynthetic performance, compatible solutes, and genes expression level as measured.

## Materials and Methods

### Plant Materials and Growth Conditions

The seeds of perennial ryegrass (Xi’an Qianwo Grass Industry Technology Co., Ltd.) were surface sterilized in 0.25% sodium hypochlorite for 10 min and then were rinsed with sterilized distilled water five times. Subsequently, the seeds were sowed to germinate in plastic cups filled with a 1:1 mixture of sand and peat soil (sterilized at 127°C in autoclave for 1 h) in a greenhouse at 25 ± 3:20 ± 3°C day:night cycle and a photoperiod of 14 h for 2 months. During the growth period, the plants were irrigated appropriately and fertilized weekly with half-strength Hoagland’s solution (Hoagland and Arnon, 1950). The materials were hand trimmed at 8 cm in height every week.

### Fungal Culture and Plant-Fungus Co-cultivation

*A. aculeatus* was used in this study and propagated in the Martin liquid medium according to our previous study (Li et al., 2017; Xie et al., 2017a). For preparation of the growth substrate, a mixture of sawdust and sand (1:3, v/v, pH = 6.5) were sieved and sterilized at 127°C in an autoclave for 60 min. Then, the mixture was separated into two halves, one of which was inoculated with the *A. aculeatus* while the half remained un-inoculated.

After 2 months of establishment, the roots of the grasses were cleansed thoroughly using sterilized ultra-purified water to remove all the soil and transplanted into plastic pots (15 cm diameter and 20 cm depth), which were filled with pre-prepared growth substances. All the materials were placed into the artificial intelligence chamber with stable conditions of 25/20°C for day/night, 14-h photoperiod, 720 μmol photons m^−2^ s^−1^ of light intensity and 66% relative humidity. The cocultivation of plants and fungus lasted for 2 weeks.

### Experimental Design and Treatments

After a successful symbiosis establishment with fungi, the plants were grouped as a same replication with similar transpiration (the transpiration rate was determined through the difference in the plant-pot system weight on a 24-h interval) and arranged in a completely randomized design with three replicates. The experiment was repeated twice to ensure that the data was reliable. Afterward, the plants were exposed to drought and heat stress. All the materials were divided into six groups, i.e., control (CK), only fungi (F), only drought stress (D), combination of drought and fungi treatment (DF), only heat stress (HT), and combination heat and fungi treatment (HTF). For drought stress, 40% of polyethylene glycol (PEG-6000) was dissolved in Hoagland nutrient solution and irrigated for 14 days. For heat stress, 25°C was the control temperature while 40°C was heat treatment, which lasted for 12 h. At the end of treatments, all the roots and leaves were harvested for various analyses.

### Measurements

#### Phytohormone Content of Leaf and Roots

Leaf and root abscisic acid (ABA) and salicylic acid (SA) concentrations were assayed according to the methods of Wang et al. (2016).

#### Chlorophyll Content and Relative Water Content of Leaf

The leaf chlorophyll content was measured according to the methods as described by Hiscox and Israelstam (1979). Briefly, the leaf chlorophyll was extracted from 0.1 g leaf sample by 15 ml dimethyl sulfoxide in the dark for 72 h. Then, the absorbance of extracting solution was measured at 645 and 663 nm using a spectrophotometer. Simultaneously, the leaf relative water content (RWC) was calculated based on the method of Barrs and Weatherley (1968): RWC = (FW − DW)/(TW − DW) × 100, where FW, DW, and TW represent fresh weight, dry weight, and turgid weight of leaf, respectively.

#### Chlorophyll a Fluorescence Transient

The Chl*a* fluorescence transient was assessed using a portable pulse-amplitude modulation (PAM) fluorometer (PAM 2500, Heinz Walz GmbH) based on the method of Chen et al. (2013). At the end of experiment, the fourth fully expanded leaves were dark-adapted for 20 min in order to close all the reaction centers of PSII and acquiring the maximal fluorescence intensity of F_M_. After a dark-adaptation for 20 min, the OJIP transients were generated by a red light of 3,000 μmol photons m^−2^ s^−1^. The chlorophyll a fluorescence emission excited by strong light pulses was monitored and then digitized between 10 and 320 μs.

#### Electrolyte Leakage

To test the cellular membrane stability, the electrolyte leakage (EL) was determined according to the method of Jespersen and Huang (2015). Briefly, a 0.1 g of fresh fully expanded leaves were cut into 0.5-cm segments and immediately placed into a 50 ml test tube filled with 15 ml deionized water, and then all tubes were incubated at room temperature for 24 h with a rotary shaker. Subsequently, the initial electrical conductivity (Ci) was recorded by a conductivity meter (JENCO-3173, Jenco Instruments, Inc., San Diego, CA, United States). To release all the leaves electrolytes, all the leaf tissues was killed in an autoclave at 121°C for 30 min. Following this, the electrical conductivity (Cmax) was determined. The EL was calculated as Ci/Cmax × 100.

#### Enzymes Activity and Lipid Peroxidation

To monitor the content of malondialdehyde (MDA) and the activity of antioxidant enzymes SOD, POD, and CAT, leaf samples (0.2 g) were grounded into powder in liquid nitrogen. Simultaneously, the roots samples (0.2 g) were also collected for determining antioxidant enzymes SOD, CAT, POD, MDA, and H_2_O_2_ content. Then, the powder was homogenized with ice-cold sodium phosphate buffer (50 mM, pH 7.8). The homogenate was centrifuged at 4°C for 20 min at 12,000 × *g* and the supernatant was collected for measuring SOD, POD, CAT, MDA, and H_2_O_2_ content.

The measurement of MDA, SOD, POD, and CAT were performed according the method of Hu et al. (2011) using spectrophotometer (UV-2600, UNICO Instruments Co., Ltd., Shanghai, China). The H_2_O_2_ content was assayed and calculated using the manufacturer protocols of hydrogen peroxide assay kit (Nanjing Jiancheng Bioengineering Institute, A064).

#### Soluble Protein and Sugar Assays

The soluble protein content was estimated based on the method of Bradford (1976), and the bovine serum albumin was used as the standard. The soluble sugar was determined according to anthranone method (Dubois et al., 1956).

#### RNA Isolation and cDNA Preparation

The total RNA of leaves was extracted using Plant Total RNA Purification Kit (Gene Mark, Taiwan) according to the manufacturer’s protocol. The purified RNA (2 μg) was reversely transcribed into cDNA based on the manufacturer’s instructions of Hifair™ II 1st Strand cDNA Synthesis Kit (YEASEN, Shanghai, China). The resultant cDNA was stored at −20°C for real time PCR analysis.

#### Primer Design and Real-Time PCR

Primer pairs of different genes were designed (Table 1) by using Primer 5 Software (PREMIER Biosoft International, Palo Alto CA, United States) and gene sequences were taken from perennial ryegrass transcriptome sequences (unpublished data) as shown in Supplementary Material. Then, the quantitative real time PCR (qTR-PCR) was performed with synthesized cDNA using gene specific primer pairs. The *eEF1A*(s) gene was used as the reference gene. For the 20 μl of reaction mixture system, 2 μl of cDNA template, 10 μl fluorescent intercalating dye SYBR green, and 0.5 μl respective gene specific primer pair were compounded with 7 μl nuclease-free water. The relative transcript level of the candidate genes was evaluated following the 2^–ΔΔCT^ method (Kenneth and Livak, 2001).

**Table 1:** Primers used for the expression of genes.

Gene name	Primer sequences (5'-3')
**HSP26.7a**	
F	TGGCTCTTGTCACACTCATCCGGAA
R	GTGAAGGTGATGGTGGAGGACGACA
**sHsp17.8-F**	
F	CGCCAAGACAGAGCAGATCAAGGCG
R	CACCGCAGCACCCAAATAAGAGCTG
**DREB1A**	
F	TCAAGAAGGAGATGAGCG
R	CGTCTCCCTGAACTTTGT
**POD47**	
F	CTCCTTGAAGTAGACGCCGTCGAAG
R	AACGTGCAGGACATGGTGGCGCTCT
**Cu/Zn-SOD**	
F	GGGAAGGTTGCTGAGCTTGATAGTG
R	CCCAGAGACAGGCAAACTCGGCAAT
**FeSOD**	
F	CTGGTAATCCCACAGCCACACGTGC
R	CAGGAGATCGACACCAACACCGACG
**eEF1A(s)**	
F	CCGTTTTGTCGAGTTTGGT
R	AGCAACTGTAACCGAACATAGC

F and R represent forward and reverse, respectively.

### Statistical Analysis

All data were expressed as mean ± SD of three replicates (*n* = 3) and were based on analysis of variance with SPSS software version 20.0 (SPSS Inc., Chicago, United States). One-way ANOVAs and Student-Newman-Keuls (SNK) test were performed to test the differences between control and treatments. The differences were considered as significant at *p* < 0.05 and indicated by different small letters.

## Results

### Phytohormone Content of Leaf and Roots

**Figure 1:**
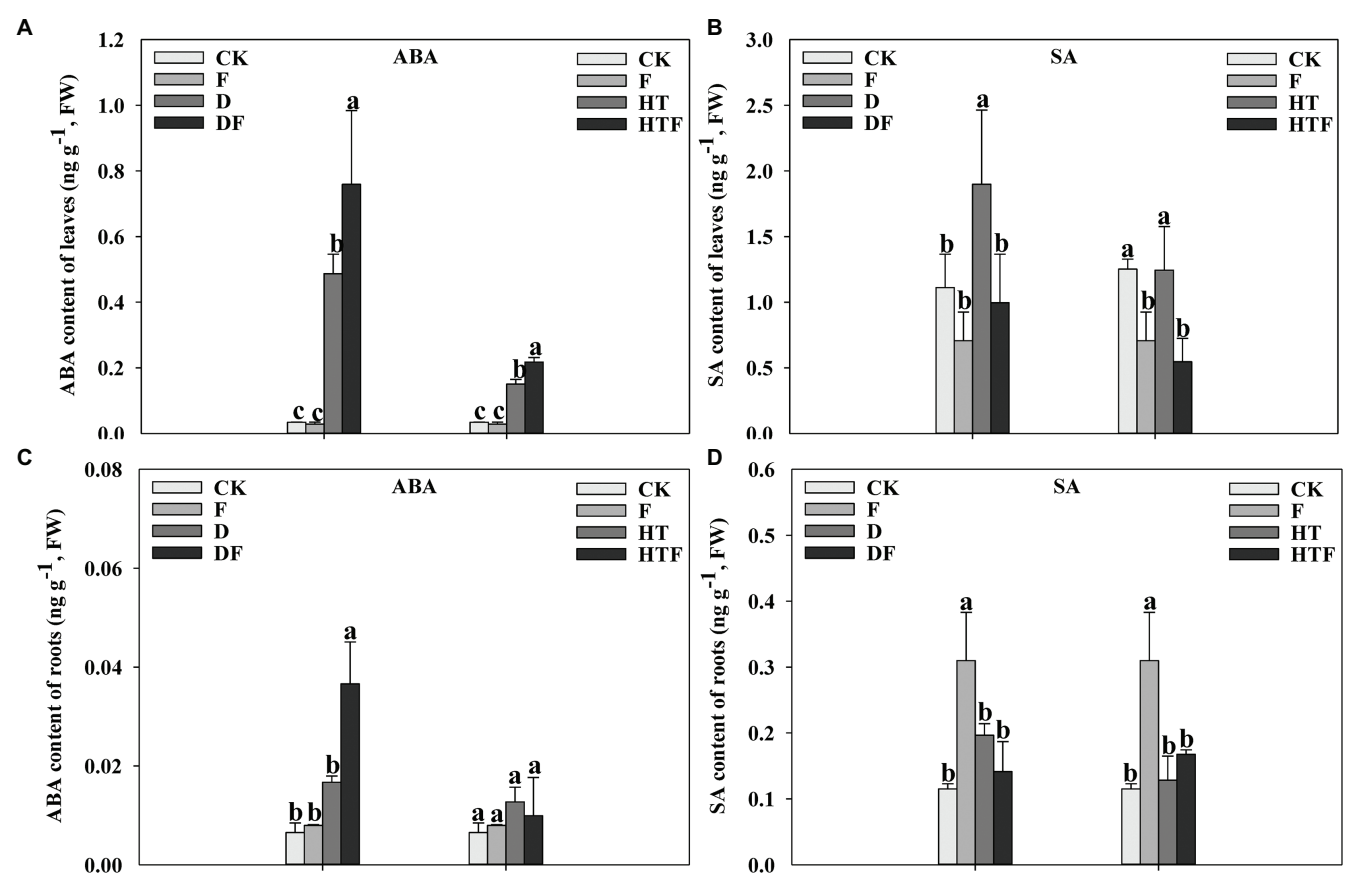
Influences of the *Aspergillus aculeatus* on abscisic acid (ABA; **A**,**C**) and salicylic acid (SA; **B**,**D**) content of perennial ryegrass leaves and roots under drought and heat stress. The differences between treatments in each parameter were detected by one-way ANOVA at *p* < 0.05 level. Bars represent mean ± SD (*n* = 3). Columns marked with same small letter (a, b, c) indicate insignificant differences between four treatment groups. CK represents control, F represents *A. aculeatus*, D represents drought, DF represents drought + *A. aculeatus*, HT represents heat, and HTF represents heat + *A. aculeatus*.

Drought and heat stress triggered an accumulation of ABA in the leaves and roots (Figure 1). Notably, ABA content in *A. aculeatus*-inoculated leaves significantly increased by 0.56-fold and 0.45-fold, respectively, when compared with non-inoculated plants exposed to drought and heat stress (Figure 1A). Simultaneously, the ABA content in the roots displayed a similar trend as leaves under drought stress (Figure 1C). In addition, drought stress increased the SA content in the leaves, which significantly decreased after the addition of *A. aculeatus*. The addition of *A. aculeatus* increased the SA content in roots. However, stress did not generate any effect on the root SA content (Figures 1B,D).

### Chlorophyll Content and Photosynthetic Efficiency

**Figure 2:**
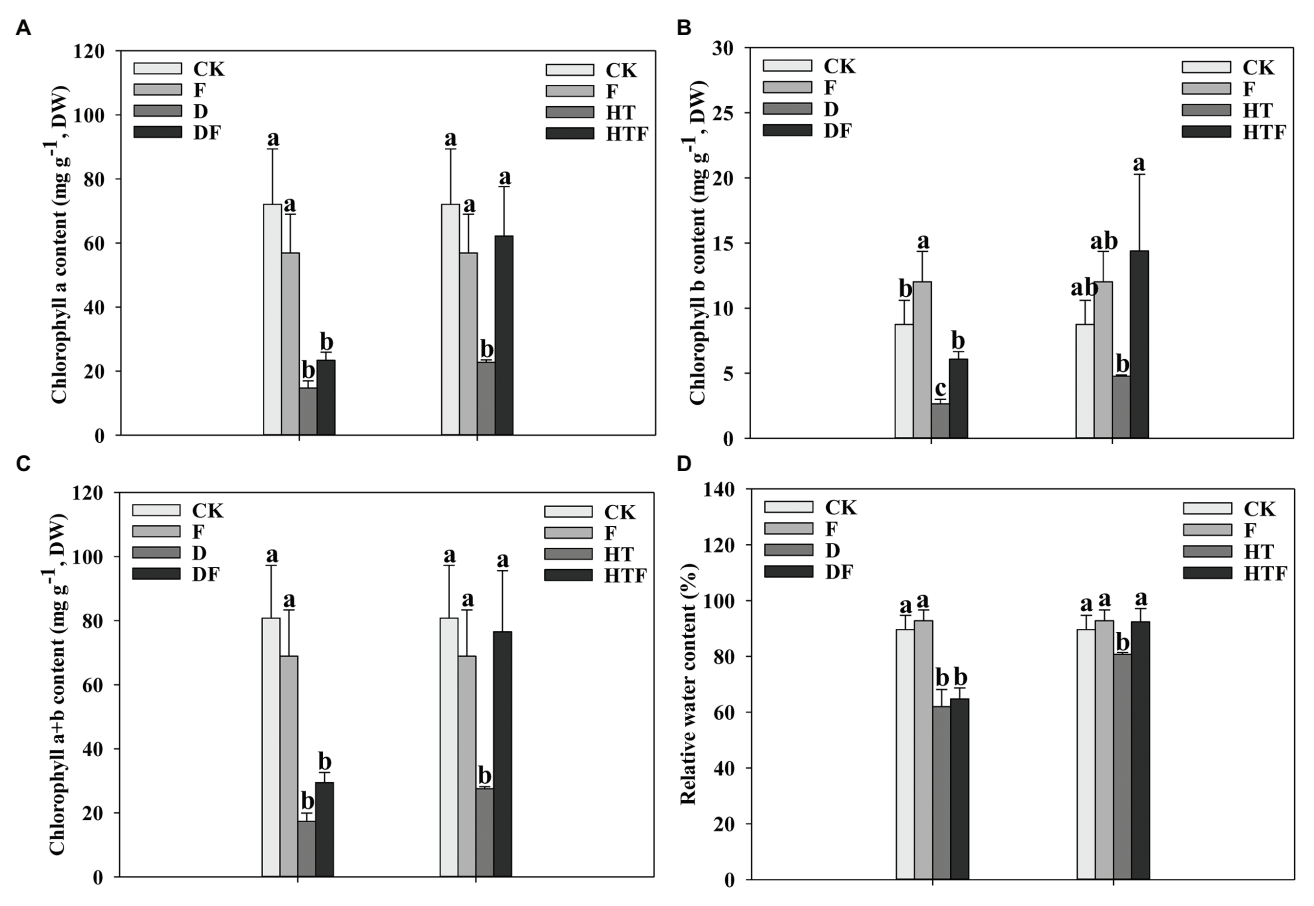
Chlorophyll a **(A)**, Chlorophyll a **(B)**, Chlorophyll a + b **(C)**, and leaf relative water content **(D)** under drought and heat stress. The differences between treatments in each parameter were detected by one-way ANOVA at *p* < 0.05 level. Bars represent mean ± SD (*n* = 3). Columns marked with same small letter (a, b, c) indicate insignificant differences between four treatment groups (*p* < 0.05). CK represents control, F represents *A. aculeatus*, D represents drought, DF represents drought + *A. aculeatus*, HT represents heat, and HTF represents heat + *A. aculeatus*.

Drought stress reduced chl *a*, chl b, and total chlorophyll contents by 4.90-fold, 3.29-fold, and 4.65-fold, respectively, relative to the control (non-stressed treatment). Synchronously, heat stress reduced chl *a*, chl b, and total chlorophyll contents by 3.17-fold, 1.83-fold, and 2.94-fold, respectively, relative to the control. Interestingly, under heat stress, the content of chl a, chl b, and total distinctly increased by 2.73-fold, 3.01-fold, and 2.78-fold, respectively, in the infected plants. Under drought stress, the content of chl b significantly increased by 2.29-fold in the infected plants. While that of chl a and total chlorophyll had no notable increase relative to the non-infected treatment (Figure 2).

**Figure 3:**
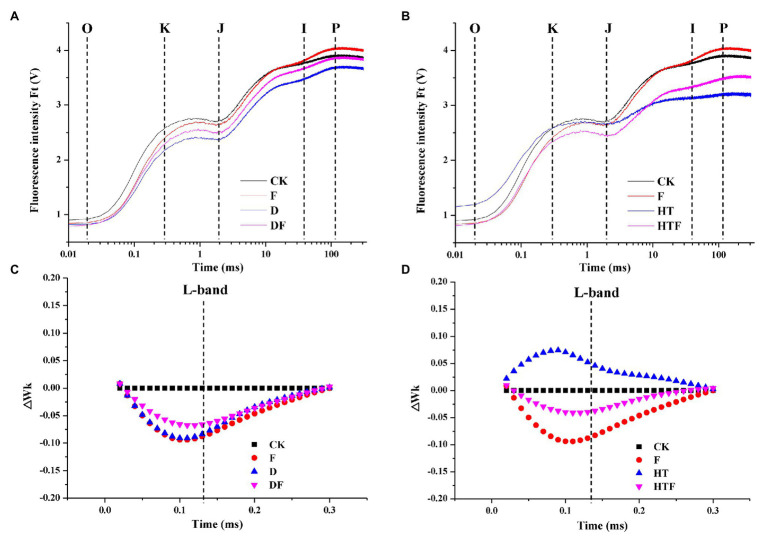
Influences of the *A. aculeatus* on chlorophyll fluorescence transients (OJIP curve; **A**,**B**) and ΔWk (**C**,**D**) in leaves of perennial ryegrass under drought and heat stress. CK represents control, F represents *A. aculeatus*, D represents drought, DF represents drought + *A. aculeatus*, HT represents heat, and HTF represents heat + *A. aculeatus*.

Application of drought and heat reduced the OJIP curves of plant leaves when compared to non-stressed plants. However, *A. aculeatus* inoculation imparted a notable increase in the OJIP fluorescence transient curves compared with their un-inoculated counterparts (Figures 3A,B). Synchronously, the stresses increased the L-band value (at about 130 μs) than the non-stressed regime. Furthermore, in *A. aculeatus*-infected plants, the value of L-band was lower than heat-infected plant under heat stress, which represented a higher energetic connectivity and stability of PSII system. (Figures 3C,D).

**Figure 4:**
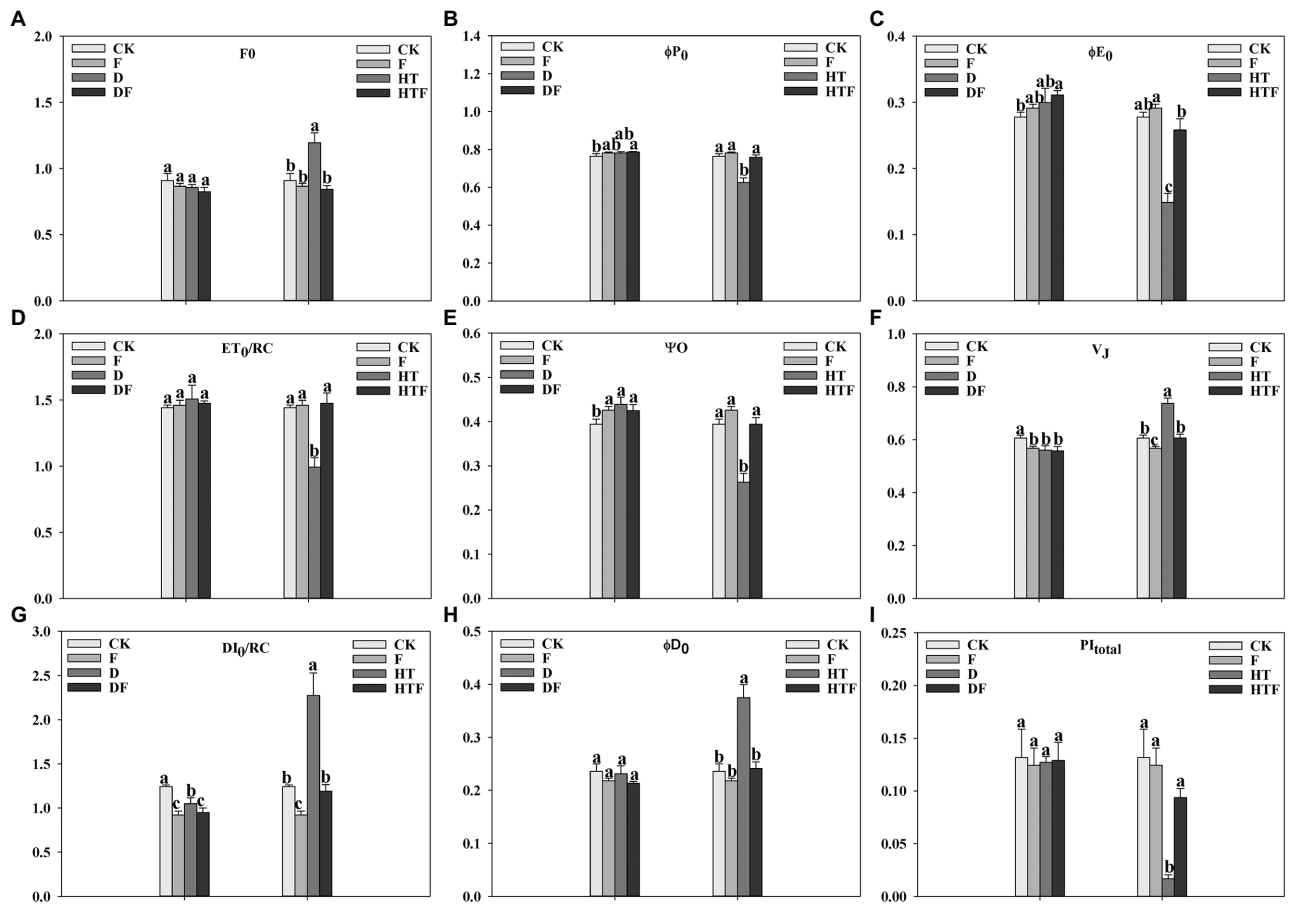
Influences of the *A. aculeatus* on photosynthetic parameters (F_0_, **A**; φP_0_, **B**; φE_0_, **C**; ET_0_/RC, **D**; Ψ_0_, **E**; V_J_, **F**; DI_0_/RC, **G**; φD_0_, **H**; PI_total_, **I**) of perennial ryegrass leaves under drought and heat stress. The differences between treatments in each parameter were detected by one-way ANOVA at *p* < 0.05 level. Bars represent mean ± SD (*n* = 3). Columns marked with same small letter (a, b, c) indicate insignificant differences between four treatment groups. CK represents control, F represents *A. aculeatus*, D represents drought, DF represents drought + *A. aculeatus*, HT represents heat, and HTF represents heat + *A. aculeatus*.

To further elucidate the regulatory mechanism of *A. aculeatus* to photosynthesis in perennial ryegrass under drought and heat stress, the fluorescence parameters were extracted and calculated from the OJIP curves. As displayed in Figure 4, the value of F_0_, V_J_, DI_0_/RC, and *φ*D_0_ had a 1.31-fold, 1.22-fold, 1.83-fold, and 1.59-fold increase, respectively, under heat stress when compared to the control. It’s worth noting that *A. aculeatus*-colonized treatment had a lower value of F_0_ (1.42-fold), V_J_ (1.22-fold), DI_0_/RC (1.91-fold), and φD_0_ (1.55-fold) under heat stress when compared with non-colonized plants. Furthermore, the value of φP_0_, φE_0_, ET_0_/RC, *Ψ*o, and PI_total_ was remarkably reduced by 1.22-fold, 1.87-fold, 1.45-fold, 1.50-fold, and 7.90-fold, respectively, under the heat regime when compared to control level. However, the inoculations of *A. aculeatus* significantly elevated the φP_0_ (1.21-fold), φE_0_ (1.74-fold), ET_0_/RC (1.48-fold), Ψo (1.50-fold), and PI_total_ (5.62-fold) values when compared to non-colonized ones.

### Membrane Lipid Peroxidation and Antioxidant Enzyme Activity of Perennial Ryegrass

**Figure 5:**
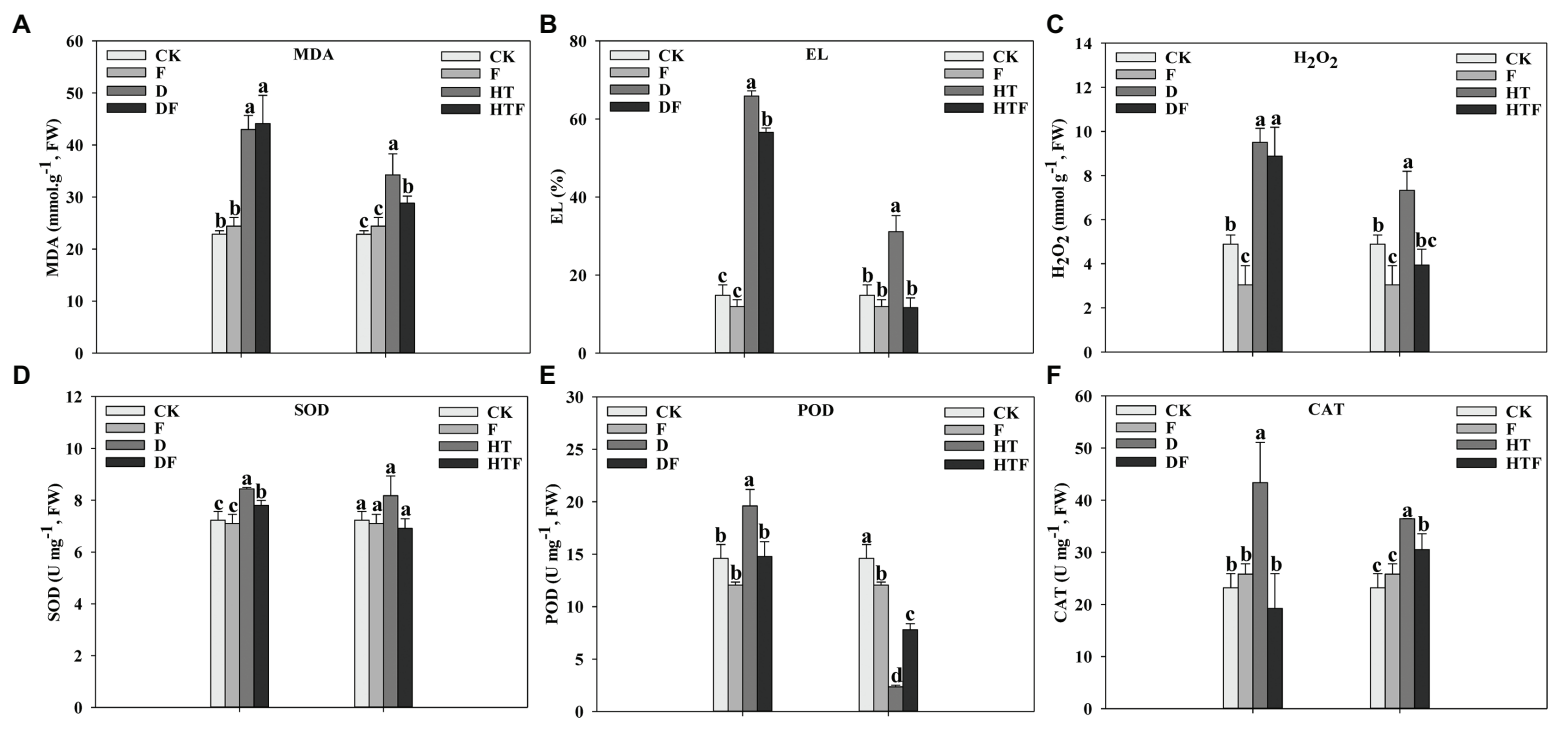
Malondialdehyde (MDA; **A**), electrolyte leakage (EL; **B**), hydrogen peroxide (H_2_O_2_; **C**), superoxide dismutase (SOD; **D**), peroxidase (POD; **E**), and catalase (CAT; **F**) content of perennial ryegrass leaves under drought and heat stress. The differences between treatments in each parameter were detected by one-way ANOVA at *p* < 0.05 level. Bars represent mean ± SD (*n* = 3). Columns marked with same small letter (a, b, c) indicate insignificant differences between four treatment groups. CK represents control, F represents *A. aculeatus*, D represents drought, DF represents drought + *A. aculeatus*, HT represents heat, and HTF represents heat + *A. aculeatus*.

To explore the protective mechanism of *A. aculeatus* on cell membrane under drought and heat stress, the leaf EL, MDA, and H_2_O_2_ levels were measured. Our results indicated that drought and heat stress greatly enhanced the levels of EL (4.44-fold and 2.10-fold, respectively), MDA (1.88-fold and 1.50-fold, respectively), and H_2_O_2_ (8.88-fold and 1.50-fold, respectively) of leaves when compared to the control. However, *A. aculeatus*-colonized leaves exhibited a decline in the EL, MDA, and H_2_O_2_ content, compared to non-colonized plants (Figures 5A–C). Overall, these observations indicated that the drought and heat stress triggered a tremendous injury on the stability of cell membranes, while *A. aculeatus* countered the injurious effects. Under drought stress, the SOD, POD, and CAT activities were strikingly elevated by 1.17-fold, 1.34-fold, and 1.87-fold, respectively, when compared to the control. Furthermore, their activities were significantly reduced in *A. aculeatus*-inoculated leaves compared to the non-inoculated under drought stress (Figures 5D–F). The CAT activity level was higher while POD activity was lower under heat stress than the control. However, inoculation of *A. aculeatus* notably decreased the CAT while increased POD activities relative to un-inoculated plants leaves (Figures 5E,F).

**Figure 6:**
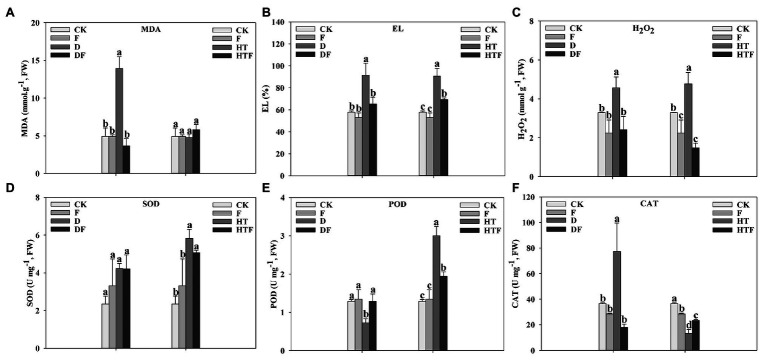
Malondialdehyde (MDA; **A**), electrolyte leakage (EL; **B**), hydrogen peroxide (H_2_O_2_; **C**), superoxide dismutase (SOD; **D**), peroxidase (POD; **E**), and catalase (CAT; **F**) content of perennial ryegrass roots under drought and heat stress. The differences between treatments in each parameter were detected by one-way ANOVA at *p* < 0.05 level. Bars represent mean ± SD (*n* = 3). Columns marked with same small letter (a, b, c) indicate insignificant differences between four treatment groups. CK represents control, F represents *A. aculeatus*, D represents drought, DF represents drought + *A. aculeatus*, HT represents heat, and HTF represents heat + *A. aculeatus*.

In addition, membrane lipid peroxidation and antioxidant activity of root were detected. As described in Figure 6, compared to the CK level (control group), drought stress notably elevated the MDA (2.84-fold), EL (1.58-fold), and H_2_O_2_ (1.39-fold) level of perennial ryegrass root, while the POD activity decreased (1.78-fold) and CAT activity increased (2.11-fold). However, after the inoculation of *A. aculeatus*, MDA, EL, and H_2_O_2_ content were significantly lower by 3.79-fold, 1.40-fold, and 1.89-fold, respectively, while POD activity was higher (1.78-fold) and CAT activity was lower (4.30-fold) than that of the non-inoculated roots. Similarly, compared with the control condition, heat stress notably elevated the level of EL and H_2_O_2_ level by 1.57-fold and 1.45-fold, respectively. Simultaneously, SOD and POD activities significantly increased by 2.48-fold and 2.34-fold, respectively, while CAT decreased by 2.78-fold. Whereas, when compared to the non-colonized ones, *A. aculeatus*-colonized remarkably declined EL (1.31-fold) and H_2_O_2_ (3.20-fold) level, while POD activity (1.54-fold) was decreased and CAT activity (1.78-fold) was increased under heat stress.

### Soluble Sugars and Proteins

**Figure 7:**
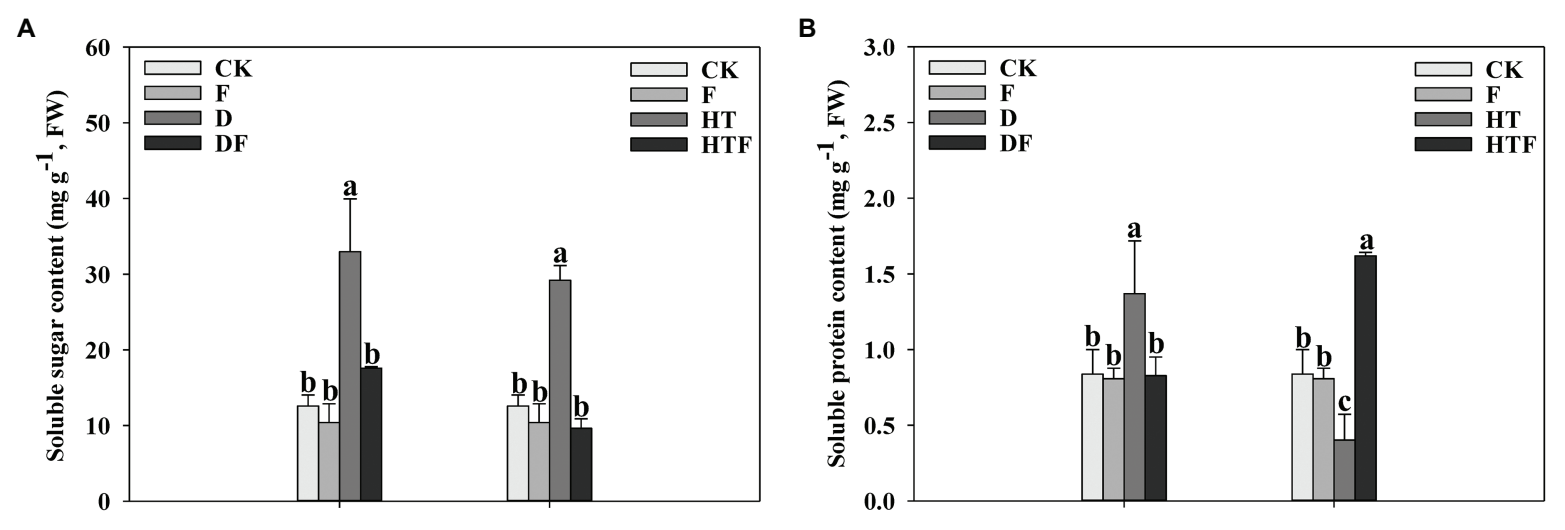
Soluble sugar **(A)** and soluble protein **(B)** content of perennial ryegrass leaves under drought and heat stress. The differences between treatments in each parameter were detected by one-way ANOVA at *p* < 0.05 level. Bars represent mean ± SD (*n* = 3). Columns marked with same small letter (a, b, c) indicate insignificant differences between four treatment groups (*p* < 0.05). CK represents control, F represents *A. aculeatus*, D represents drought, DF represents drought + *A. aculeatus*, HT represents heat, and HTF represents heat + *A. aculeatus*.

As depicted in Figure 7, drought caused a distinctive accumulation of soluble sugar (2.62-fold) and soluble protein (1.63-fold), when compared to the control. For *A. aculeatus*-colonized plant, the soluble sugar and soluble protein content exhibited an obvious decline (0.53-fold and 0.60-fold, respectively) when compared with non-colonized leaves. Moreover, heat stress causes a higher accumulation of soluble sugar, while lower soluble protein content than the control. Nevertheless, in *A. aculeatus*-infected plants, a strong decline in the soluble sugars level was detectable, while an obvious increase of the soluble protein compared to uninfected ones.

### Relative Expression Levels of Genes

**Figure 8:**
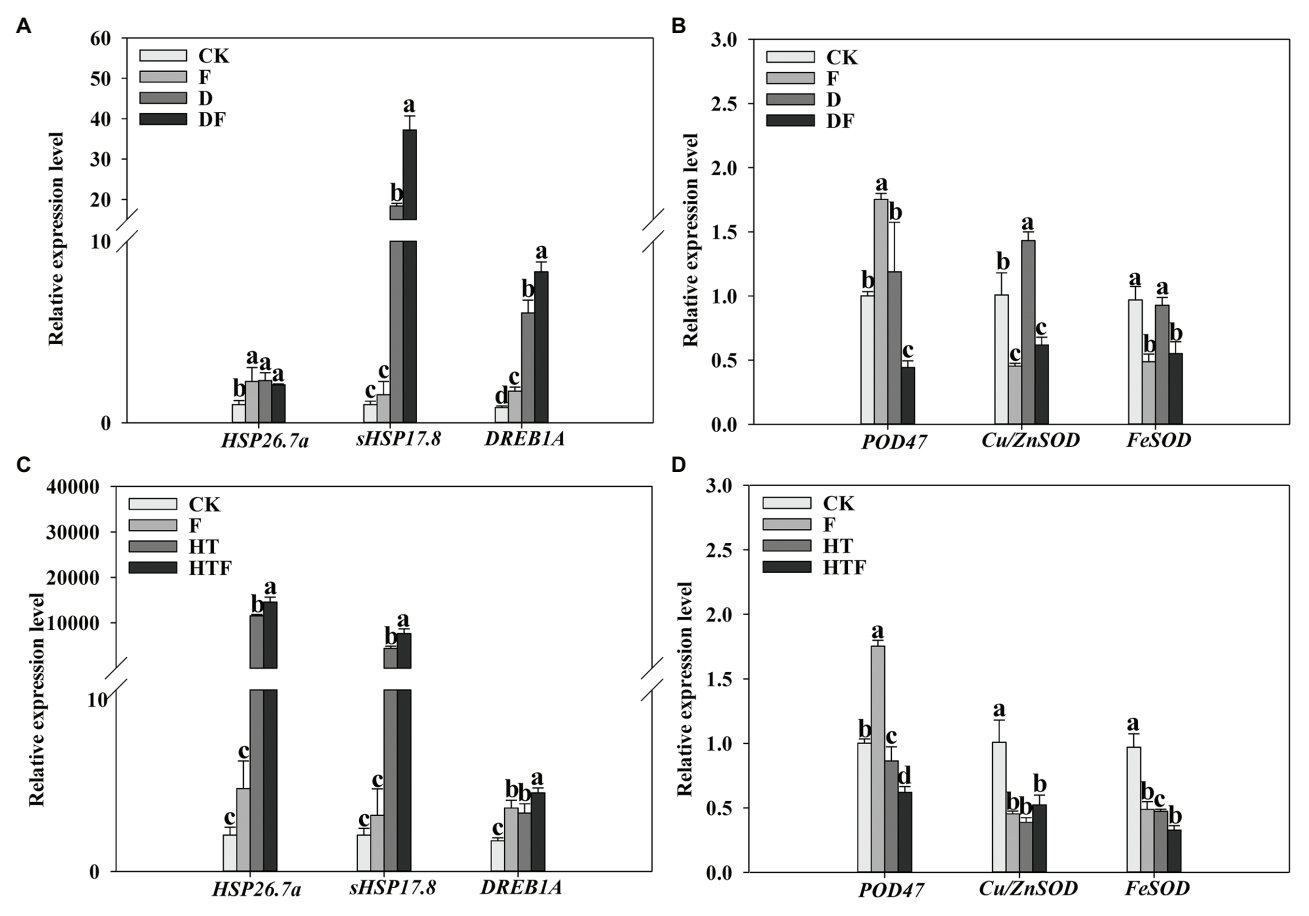
Influences of the *A. aculeatus* on gene expression level (*HSP26.7a*, *sHSP17.8*, and *DREB1A*, **A**,**C**; *POD47*, *Cu/ZnSOD*, and *FeSOD*, **B**,**D**) of perennial ryegrass leaves under drought and heat stress. The differences between treatments in each parameter were detected by one-way ANOVA at *p* < 0.05 level. Bars represent mea ± SD (*n* = 3). Columns marked with same small letter (a, b, c) indicate insignificant differences between four treatment groups. CK represents control, F represents *A. aculeatus*, D represents drought, DF represents drought + *A. aculeatus*, HT represents heat, and HTF represents heat + *A. aculeatus*.

The expression levels of *HSP26.7a*, *sHSP17.8*, *DREB1A*, *POD47*, *Cu/ZnSOD*, and *FeSOD* were analyzed in shoot tissues as shown in Figure 8. Drought stress induced an upregulation of genes *HSP26.7a*, *sHSP17.8*, *DREB1A*, and *Cu/ZnSOD* genes by 2.32-fold, 18.35-fold, 7.18-fold and 1.42-fold, respectively, when compared to the control (Figures 8A,B). Interestingly, under drought exposure, the expression levels of *sHSP17.8* (2.03-fold) and *DREB1A* (1.37-fold) were obviously elevated while *POD47*, *Cu/ZnSOD*, and *FeSOD* were significantly decreased by 2.29-fold, 2.32-fold, and 1.67-fold, respectively, in fungi-colonized plants compared to those non-colonized regimes. Simultaneously, the *HSP26.7a*, *sHSP17.8*, and *DREB1A* were 11501.18-fold, 4375.44-fold, and 1.92-fold upregulated under heat stress when compared to the control condition. It is noteworthy that the expression levels of *HSP26.7a* (1.26-fold), *sHSP17.8* (1.75-fold), and *DREB1A* (1.34-fold) were remarkably enhanced, while *POD47* and *FeSOD* were significantly decreased by 1.39-fold and 1.45-fold, respectively, in *A. aculeatus*-infected plants when compared with heat treatment alone (Figure 8).

## Discussions

Plants respond to adverse environmental conditions *via* manipulating the metabolic activity of antioxidants, osmolytes, and phytohormone (Kumar, 2012; Hasanuzzaman et al., 2013). Also, stress-responsive hormone ABA plays a pivotal role in plants response to drought and heat stress (Zhang et al., 2006; Khan et al., 2012; Hasanuzzaman et al., 2013). Consistent with Khan et al. (2012) and Ibrahim et al. (2019) findings, our results showed that drought and heat remarkably enhanced the ABA content of plant leaves compared with the corresponding controls. Furthermore, our findings also reinforce the notion that the increase of plant ABA content was accompanied by the inoculation of beneficial microbes (Sannazzaro et al., 2006). In parallel to the finding, our study revealed a higher concentration of ABA in *A. aculeatus*-inoculated leaves than non-inoculated plants under drought and heat stresses. Besides, under drought stress, ABA content in the roots showed a similar change trend with leaves. Therefore, these observations might suggest that the accumulation of ABA induced by *A. aculeatus* inoculation could confer resistance to drought and stresses *via* maintaining a higher conductance and water condition of host plants. On the other hand, a previous study illustrated that SA could enhance plant performance by inducing systemic resistance to biotic and abiotic stresses (Pozo and Azcónaguilar, 2007). In our results, the SA content was distinctly elevated under drought stress. Nevertheless, in inoculated plants leaves, the SA content was lower than non-inoculated leaves. Taken together, our results indicated that variational ABA and consequent SA-signaling might be one of the mechanisms by which *A. aculeatus* can induce plant tolerance to drought and heat stresses.

Chlorophyll *a* fluorescence is considered to be a powerful and reliable non-invasive tool for exploring plant photosynthetic efficiency and the function of PSII under adverse environmental conditions (Kalaji et al., 2011; Chen et al., 2013). In our study, we performed a comprehensive investigation on the PSII photochemistry of perennial ryegrass subjected to heat stress. Chen et al. (2013) reported that photosynthesis as one of the most sensitive processes to heat stress was easily damaged by high temperature. In agreement with their study, our results displayed a remarkable decline in the OJIP fluorescence transient curves of heat-exposed leaves. To further shed light on the PSII reaction centers activity, photosynthetic parameters, such as F_0_, V_J_, *φ*P_0_ (F_V_/Fm), φE_0_, ET_0_/RC, *Ψ*_O_, φD_0_, DI_0_/RC, and PI_total_, were used for in-depth analyses. In our observations, the φP_0_, φE_0_, ET_0_/RC, Ψo, and PI_total_ of plant leaves were dramatically decreased under heat stress compared to the control. Among these parameters, reductions in the φE_0_, φP_0_, and Ψo were accompanied by a decreased quantum yield of the electron transport flux from Quinone A to Quinone B, reduced maximum quantum yield for primary photochemistry and declined efficiency that a trapped exciton can φmove an electron into the electron transport chain beyond Q_A-_, which indicates that high temperature inhibited electron transfer on the PSII receptor side. In contrast, under heat stress, the F_0_, V_J_, DI_0_/RC, and *φ*D_0_ were remarkably increased. The increase of F_0_ value was triggered by the physical separation of the PSII reaction centers from their combined pigment antennae leading in the blocked energy transportation to the PSII traps (Srivastava et al., 1997). Meanwhile, the variable fluorescence of V_J_ reflects the electron transfer characteristics from Q_A_ to Q_B_ on the PSII electron acceptor side (Strasser, 1997) and closure degree of reactive center when chlorophyll fluorescence reaches J phase (Force et al., 2003). In our results, heat stress causes an increase of V_J_ value, which suggests that heat stress inhibits electron transfer from Q_A_ to Q_B_, leading to accumulation of Q_A_-, and at the same time, indicating partial closure of active reaction centers. Furthermore, extensive studies have confirmed that heat stress could induce inactivation of oxygen-evolving complex (OEC), inhibition of electron transport, and decline in PSII photochemical efficiency (Strasser, 1997; De Ronde et al., 2004; Wahid et al., 2007). In our results, it is noteworthy that the L-band shows a remarkable increase under heat treatment, which explains the behavior of K-step in OJIP curves. The appearance of K-step is a distinctive characteristic in fluorescence rise kinetics when leaves subjected to heat stress were ascribed to the destruction of OEC triggered by releasing manganese cluster (Strasser et al., 2004). Chen et al. (2013) findings reinforce this observation. Therefore, these changes of photosynthetic parameters collectively suggested that heat stress aggravated the damage of thylakoid membrane and OEC, declined the efficiency of photosynthetic electron transport rate and increased the energy dissipation of reaction center.

Studies have reported that mycorrhiza fungi could mitigate the negative effects of abiotic stress on PSII reaction centers and photosynthetic performance (Zhu et al., 2011; Xie et al., 2017a; Duc et al., 2018; Khalid et al., 2018). Here, the protective effects of *A. aculeatus* on OJIP fluorescence transient of perennial ryegrass subjected to heat stress are shown in Figures 2, 3. The inoculation of *A. aculeatus* alleviated the detrimental effects of heat stress on PSII reaction centers corresponding to increased *φ*P_0_, φE_0_, ET_0_/RC, *Ψ*o, and PI_total_ and decreased F_0_, V_J_, DI_0_/RC, and φD_0_, diminished K-step and lower L-band, compared to corresponding non-inoculated plant. The value of related electron transport parameters on the PSII receptor side (φE_0_, φP_0_, and Ψo) had a prominent increase in inoculated-plant leaves, when compared to non-inoculated plant exposed to heat stress, indicating that *A. aculeatus* promoted electron transport on the PSII receptor side. In addition, in our results, inoculation of *A. aculeatus* triggered the decrease of V_J_ value under heat stress, when compared to non-inoculated plant, which suggests that *A. aculeatus* promotes the electron transfer from Q_A_ to Q_B_. At the same time, the *A. aculeatus*-induced increase in Ψo further confirmed fungi enhanced reoxidation capacity of Q_A_-, thereby promoting electron transfer after Q_A_-. Similarly, Zhu et al. (2011) have documented that mycorrhizal fungi reduced damage induced by high temperature treatment, which accompanying with enhanced chlorophyll and carotenoid content, value of F_V_/Fm (φP_0_) and PSII reaction activity. Taken together, these results implied that the inoculation of *A. aculeatus* modulates the imbalance between light absorption and utilization caused by high temperature though enhancing the efficiency of photosynthetic electron transport rate and reducing the energy dissipation of PSII reaction centers.

To explore on *A. aculeatus*-mediated protective mechanism of ryegrass against drought and heat stress, the lipid peroxidation indexes, such as EL, MDA, and H_2_O_2_, were assessed. Our results showed that drought and heat significantly increase the EL, MDA, and H_2_O_2_ level when compared to control condition. Concurrent to this study, Bi et al. (2016) found that drought and high temperature increased the EL, MDA, and H_2_O_2_ content in tall fescue. In our findings, it should be noted that in *A. aculeatus*-inoculated plants, the membrane damage and lipid peroxidation were dramatically alleviated, which was accompanied by a decline in EL, MDA, and H_2_O_2_ content. Consistent with our findings, Khalid et al., (2018) observed that the application of *P. indica* availably modulated the integrity of the cell membrane when plants were exposed to unfavorable conditions. Taken together, our results and previous observations suggest that *A. aculeatus* acting as a “health insurance” played a crucial role in maintaining cell membrane functions and ameliorating lipid peroxidation, especially under drought and heat stress. At the same time, alongside ROS generation, plants have established a complex antioxidative detoxification system (Mittler, 2002; Janda et al., 2003; Harsh et al., 2016). Here, the SOD, POD, and CAT activities were decreased by *A. aculeatus* under drought stress. One explanation for this phenomenon may be that *A. aculeatus* alleviated the oxidative damage caused by the stresses, and on the other hand, H_2_O_2_ overproduction was tackled by the action of GSH, where the H_2_O_2_ was reduced to harmless H_2_O. Previous studies have demonstrated that GSH is an important antioxidant and signal molecule that plays an essential role in response to and stress tolerance (Kok and Oosterhuis, 1983; Janda et al., 2003). Our previous study confirmed that *A. aculeatus* elevated GSH concentration under salt stress (Xie et al., 2017a). Different from drought stress, *A. aculeatus* induced the increase of POD activity and the decrease of CAT activity under heat stress. These results implied heat stress alters the activity of ROS scavenging enzymes and induced a compensation mechanism. Therefore, although the POD activity decreases, plants could remove more ROS by increasing the CAT activity under heat stress. After inoculation of *A. aculeatus*, the damage of heat stress to perennial ryegrass was alleviated, this was manifested as the increased POD activity and the decreased CAT activity. Overall, our results suggested that enhanced tolerance to drought and heat stress can be associated with remission of lipid peroxidation in *A. aculeatus*-colonized plants.

Plants cope with changing environmental conditions by altering the expression level of stress-related genes (Krasensky and Jonak, 2012). HSPs are involved in a variety of environmental stressors (Yee-Yung et al., 2007; Timperio et al., 2009; Ayako et al., 2010). In line with these findings here, we observe that *sHSP17.8* was dramatically upregulated under drought and heat stress, compared to control condition. Intuitively, under drought and heat stress, the expression level of *sHSP17.8* was higher in *A. aculeatus*-inoculated leaves than non-inoculated regimes. In addition, many literatures confirmed that DREB transcription factors are induced by a variety of abiotic stresses such as heat, drought, salt, and low temperature stresses (Liu et al., 1998; Dubouzet et al., 2003; Chen et al., 2007). In our results, heat and drought stress induced the upregulation of *DREB1A*. After inoculation of *A. aculeatus*, the *DREB1A* expression level was further enhanced. Based on the above results, we suggested that *A. aculeatus* could enhance the plant’s adaptability to heat and drought stress by increasing the expression of *HSP17.8* and *DREB1A*. In addition to scavenging the reactive oxygen intermediates (ROIs) generated by stresses, the expression of ROI-scavenging enzymes genes was induced under stress (Dat et al., 2000; Mittler, 2002). Our findings showed that drought stress induced an upregulation of *Cu/ZnSOD*, which was associated with increased of SOD activity when compared to control level. Notably, it was observed that *A. aculeatus* triggered the downregulation of *Cu/ZnSOD* and decreased of SOD activity in inoculated-plant, compared with non-inoculated ones under drought stress. Similarly, under heat stress, the downregulated expression level of *POD47* and *FeSOD* were further enhanced in inoculated regimes, compared to non-inoculated ones. Taking these results together, *A. aculeatus* may confer drought and heat tolerance to perennial ryegrass by altering the expression of drought and heat-related genes.

## Conclusion

In summary, by using perennial ryegrass plants and mutualistic *A. aculeatus*, we provide the evidence for the fungal alleviatory effects to the adverse effects of drought and heat stress. Our results indicate that *A. aculeatus* confers tolerance to drought and heat stress by altering physiological and biochemical indexes as well as gene expression levels of plants. According to our results, we propose four important *A. aculeatus*-mediated mechanisms of perennial ryegrass response to drought and heat stress: (1) *A. aculeatus* can regulate hormone homeostasis, (2) reduced the damage to photosynthetic system of plants induced by drought and heat stress, (3) reduced oxidative damage of plants induced by drought and heat stress, (4) altered gene expression levels related to drought and heat stress.

## Data Availability Statement

Requests to access these datasets should be directed to XL, lixiaoning0724@126.com.

## AuthOr Contributions

XL conceived the experiments and wrote the manuscript. XL and GW performed the experiments and analyzed the data. CZ and TZ cultivated the experimental materials. EA revised the manuscript. YX and JF guided this experiment. All authors contributed to the article and approved the submitted version.

### Conflict of Interest

The authors declare that the research was conducted in the absence of any commercial or financial relationships that could be construed as a potential conflict of interest.

## Abbreviations

ABA: Abscisic acid
CAT: Catalase
DI_0_/RC: Dissipated energy flux per reaction centers
EL: Electrolyte leakage
ET_0_/RC: Electron transport flux per PSII reaction center
F_0_: Minimal recorded fluorescence intensity
H_2_O: Hydrogen peroxide
HSPs: Heat shock proteins
OH^−^: Hydroxyl
O_2_^−^: Superoxide radicals
PI_total_: Performance index for energy conservation from exciton to the reduction of PSI end acceptors
POD: Peroxidase
PSII: Photosystem II
ROS: Reactive oxygen species
V_J_: Relative variable fluorescence at the J-step
SA: Salicylic acid
SOD: Superoxide dismutase
*φ*P_0_: Maximum quantum yield for primary photochemistry, namely F_V_/F_M_
φE_0_: Quantum yield of the electron transport flux from Quinone A to Quinone B
*Ψ*_O_: Efficiency that a trapped exciton can move an electron into the electron transport chain beyond Q_A-_
φD_0_: Quantum yield (at t = 0) of energy dissipation

## References

[ref1] Al-Khatib K. Paulsen G. M. (1999). High-temperature effects on photosynthesis processes in temperate and tropical cereals. Crop Sci. 39, 119–125. 10.2135/cropsci1999.0011183X003900010019x

[ref2] Apel K. Hirt H. (2004). Reactive oxygen species: metabolism, oxidative stress, and signal transduction. Annu. Rev. Plant Biol. 55, 373–399. 10.1146/annurev.arplant.55.031903.141701, PMID: 15377225

[ref3] Ayako N. Yukinori Y. Eriko Y. Takanori M. Kazuya Y. Shigeru S. (2010). Arabidopsis heat shock transcription factor A2 as a key regulator in response to several types of environmental stress. Plant J. 48, 535–547. 10.1111/j.1365-313X.2006.02889.x, PMID: 17059409

[ref4] Baltruschat H. Fodor J. Harrach B. D. Niemczyk E. Barna B. Gullner G.. (2008). Salt tolerance of barley induced by the root endophyte *Piriformospora indica* is associated with a strong increase in antioxidants. New Phytol. 180, 501–510. 10.1111/j.1469-8137.2008.02583.x, PMID: 18681935

[ref5] Barrs H. D. Weatherley P. E. (1968). A re-examination of the relative turgidity technique for estimating water deficits in leaves. Aust. J. Biol. Sci. 15, 413–428. 10.1071/BI9620413

[ref6] Berry J. Björkman O. (1980). Photosynthetic response and adaptation to temperature in higher plants. Annu. Rev. Plant Physiol. 31, 491–543. 10.1146/annurev.pp.31.060180.002423

[ref7] Bi A. Fan J. Hu Z. Wang G. Amombo E. Fu J.. (2016). Differential acclimation of enzymatic antioxidant metabolism and photosystem II photochemistry in tall fescue under drought and heat and the combined stresses. Front. Plant Sci. 7:453. 10.3389/fpls.2016.00453, PMID: 27148288 PMC4830848

[ref8] Bilger W. Schreiber U. Lange O. L. (1987). “Chlorophyll fluorescence as an indicator of heat induced limitation of photosynthesis in *Arbutus unedo* L” in Plant Responses to Stress. Functional Analysis in Mediterranean Ecosystems. eds. Tenhunen J. D. Catarino F. M. Lange O. L. Oechel W. C. (Berlin: Springer-Verlag), 391–399.

[ref9] Bradford M. M. (1976). A rapid and sensitive method for the quantitation of microgram quantities of protein utilizing the principle of protein-dye binding. Anal. Biochem. 72, 248–254. 10.1016/0003-2697(76)90527-3, PMID: 942051

[ref10] Chao S. Johnson J. M. Cai D. Sherameti I. Oelmüller R. Lou B. (2010). Piriformospora indica confers drought tolerance in Chinese cabbage leaves by stimulating antioxidant enzymes, the expression of drought-related genes and the plastid-localized CAS protein. J. Plant Physiol. 167, 1009–1017. 10.1016/j.jplph.2010.02.013, PMID: 20471134

[ref11] Chen K. Chen L. Fan J. Fu J. (2013). Alleviation of heat damage to photosystem II by nitric oxide in tall fescue. Photosynth. Res. 116, 21–31. 10.1007/s11120-013-9883-5, PMID: 23832593

[ref12] Chen M. Wang Q. Y. Cheng X. G. Xu Z. S. Li L. C. Ye X. G.. (2007). *Gm DREB2*, a soybean DRE-binding transcription factor, conferred drought and high-salt tolerance in transgenic plants. Biochem. Biophys. Res. Commun. 353, 299–305. 10.1016/j.bbrc.2006.12.027, PMID: 17178106

[ref13] Dat J. Vandenabeele S. Vranová E. Van Montagu M., Inzé D. Van Breusegem F. (2000). Dual action of the active oxygen species during plant stress responses. Cell. Mol. Life Sci., 57, 779–795. 10.1007/s000180050041, PMID: 10892343 PMC11147059

[ref14] De Ronde J. A. Cress W. A. Krüger G. H. Strasser R. J. Van S. J. (2004). Photosynthetic response of transgenic soybean plants, containing an Arabidopsis *P5CR* gene, during heat and drought stress. J. Plant Physiol. 161, 1211–1224. 10.1016/j.jplph.2004.01.014, PMID: 15602813

[ref15] Dubois M. Gilles K. A. Hamilton J. K. Rebers P. A. Smith F. (1956). Colorimetric method for determination of sugars and related substances. Anal. Chem. 28, 350–356. 10.1021/ac60111a017

[ref16] Dubouzet J. G. Sakuma Y. Ito Y. Kasuga M. Dubouzet E. G. Miura S.. (2003). *OsDREB* genes in rice, *Oryza sativa* L., encode transcription activators that function in drought-, high-salt‐ and cold-responsive gene expression. Plant J. 33, 751–763. 10.1046/j.1365-313X.2003.01661.x, PMID: 12609047

[ref17] Duc N. H. Csintalan Z. Posta K. (2018). Arbuscular mycorrhizal fungi mitigate negative effects of combined drought and heat stress on tomato plants. Plant Physiol. Biochem. 132, 297–307. 10.1016/j.plaphy.2018.09.011, PMID: 30245343

[ref18] Feller U. (2007). Photosynthetic performance and water relations in young pubescent oak (*Quercus pubescens*) trees during drought stress and recovery. New Phytol. 174, 799–810. 10.1111/j.1469-8137.2007.02047.x, PMID: 17504463

[ref19] Force L. Critchley C. Rensen J. J. S. (2003). New fluorescence parameters for monitoring photosynthesis in plants. Photosynth. Res. 78, 17–33. 10.1023/A:1026012116709, PMID: 16245061

[ref20] Hannaway D. B. Fransen S. Cropper J. B. Teel M. Chaney M. Griggs T. D.. (1999). Perennial ryegrass (*Lolium perenne* L.). Methods Mol. Biol. 344:55. 10.1385/1-59745-131-2:55, PMID: 17033051

[ref21] Harsh A. Sharma Y. K. Joshi U. Rampuria S. Singh G. Kumar S.. (2016). Effect of short-term heat stress on total sugars, proline and some antioxidant enzymes in moth bean (*Vigna aconitifolia*). Ann. Agric. Sci. 61, 57–64. 10.1016/j.aoas.2016.02.001

[ref22] Hasanuzzaman M. Nahar K. Alam M. M. Roychowdhury R. Fujita M. (2013). Physiological, biochemical, and molecular mechanisms of heat stress tolerance in plants. Int. J. Mol. Sci. 14, 9643–9684. 10.3390/ijms14059643, PMID: 23644891 PMC3676804

[ref23] Hiscox J. D. Israelstam G. F. (1979). A method for the extraction of chlorophyll from leaf tissue without maceration. Can. J. Public Health 57, 1332–1334. 10.1139/b79-163

[ref24] Hoagland D. R. Arnon D. I. (1950). The water-culture method for growing plants without soil. Vol. 347. Berkeley, CA: College of Agriculture, University of California, 357–359.

[ref25] Hu T. Li H. Y. Zhang X. Z. Luo H. J. Fu J. M. (2011). Toxic effect of NaCl on ion metabolism, antioxidative enzymes and gene expression of perennial ryegrass. Ecotoxicol. Environ. Saf. 74, 2050–2056. 10.1016/j.ecoenv.2011.07.013, PMID: 21813179

[ref26] Ibrahim W. Zhu Y. M. Chen Y. Qiu C. W. Zhu S. J. Wu F. B. (2019). Genotypic differences in leaf secondary metabolism, plant hormones and yield under alone and combined stress of drought and salinity in cotton genotypes[J]. Physiol. Plant. 165, 343–355. 10.1111/ppl.12862, PMID: 30367694

[ref27] Iwayainoue M. Matsui R. Fukuyama M. (2004). Cold‐ or heat-tolerance of leaves and roots in perennial ryegrass determined by 1H-NMR. Plant Prod. Sci. 7, 118–128. 10.1626/pps.7.118

[ref28] Janda T. Szalai G. Rios-Gonzalez K. Veisz O. Páldi E. (2003). Comparative study of frost tolerance and antioxidant activity in cereals. Plant Sci. 164, 301–306. 10.1016/S0168-9452(02)00414-4

[ref29] Jespersen D. Huang B. (2015). Proteins associated with heat-induced leaf senescence in creeping bentgrass as affected by foliar application of nitrogen, cytokinins, and an ethylene inhibitor. Proteomics 15, 798–812. 10.1002/pmic.201400393, PMID: 25407697

[ref30] Jiang Y. Huang B. (2001). Drought and heat stress injury to two cool-season turfgrasses in relation to antioxidant metabolism and lipid peroxidation. Crop Sci. 41, 436–442. 10.2135/cropsci2001.412436x

[ref31] Kalaji H. M. Govindjee Bosa K. Kościelniak J. Żuk-Gołaszewska K. (2011). Effects of salt stress on photosystem II efficiency and CO_2_ assimilation of two Syrian barley landraces. Environ. Exp. Bot. 73, 64–72. 10.1016/j.envexpbot.2010.10.009

[ref32] Kenneth J. Livak T. D. (2001). Analysis of relative gene expression data using rea l—time quantitative PCR and the 2-ct method. Methods 25, 402–408. 10.1006/meth.2001.1262, PMID: 11846609

[ref33] Khalid M. Hassani D. Liao J. Xiong X. Bilal M. Huang D. (2018). An endosymbiont Piriformospora indica reduces adverse effects of salinity by regulating cation transporter genes, phytohormones, and antioxidants in *Brassica campestris ssp. chinensis*. Environ. Exp. Bot. 153, 89–99. 10.1016/j.envexpbot.2018.05.007

[ref34] Khan A. L. Hamayun M. Waqas M. Kang S. M. Kim Y. H. Kim D. H.. (2012). Exophiala sp. LHL08 association gives heat stress tolerance by avoiding oxidative damage to cucumber plants. Biol. Fertil. Soils 48, 519–529. 10.1007/s00374-011-0649-y

[ref35] Kok L. J. D. Oosterhuis F. A. (1983). Effects of frost-hardening and salinity on glutathione and sulfhydryl levels and on glutathione reductase activity in spinach leaves. Physiol. Plant. 58, 47–51. 10.1111/j.1399-3054.1983.tb04141.x

[ref36] Krasensky J. Jonak C. (2012). Drought, salt, and temperature stress-induced metabolic rearrangements and regulatory networks. J. Exp. Bot. 63, 1593–1608. 10.1093/jxb/err460, PMID: 22291134 PMC4359903

[ref37] Kumar S. (2012). Abscisic acid induces heat tolerance in chickpea (*Cicer arietinum* L.) seedlings by facilitated accumulation of osmoprotectants. Acta Physiol. Plant. 34, 1651–1658. 10.1007/s11738-012-0959-1

[ref38] Ladjal M. Epron D. Ducrey M. (2000). Effects of drought preconditioning on thermotolerance of photosystem II and susceptibility of photosynthesis to heat stress in cedar seedlings. Tree Physiol. 20, 1235–1241. 10.1093/treephys/20.18.1235, PMID: 12651486

[ref40] Li X. Han S. Wang G. Liu X. Amombo E. Xie Y.. (2017). The fungus *Aspergillus aculeatus* enhances salt-stress tolerance, metabolite accumulation, and improves forage quality in perennial ryegrass. Front. Microbiol. 8:1664. 10.3389/fmicb.2017.01664, PMID: 28936200 PMC5595160

[ref39] Li G. C. Werb Z. (1982). Correlation between synthesis of heat shock proteins and development of thermotolerance in Chinese hamster fibroblasts. PNAS 79, 3218–3222. 10.1073/pnas.79.10.3218, PMID: 6954473 PMC346386

[ref41] Liu Q. Kasuga M. Sakuma Y. Abe H. Miura S. Yamaguchi-Shinozaki K.. (1998). Two transcription factors, *DREB1* and *DREB2*, with an EREBP/AP2 DNA binding domain separate two cellular signal transduction pathways in drought‐ and low-temperature-responsive gene expression, respectively, in Arabidopsis. Plant Cell 10, 1391–1406. 10.1105/tpc.10.8.1391, PMID: 9707537 PMC144379

[ref42] Mittler R. (2002). Oxidative stress, antioxidants and stress tolerance. Trends Plant Sci. 7, 405–410. 10.1016/S1360-1385(02)02312-9, PMID: 12234732

[ref43] Mohammadkhani N. Heidari R. (2014). Effects of drought stress on soluble proteins in two maize varieties. Turk. J. Biol. 32, 23–30.

[ref44] Narsian V. Patel H. H. (2000). *Aspergillus aculeatus* as a rock phosphate solubilizer. Soil Biol. Biochem. 32, 559–565. 10.1016/S0038-0717(99)00184-4

[ref45] Niyogi K. K. (1999). Photoprotection Revisited: genetic and molecular approaches. Annu. Rev. Plant Physiol. Plant Mol. Biol. 50, 333–359. 10.1146/annurev.arplant.50.1.333, PMID: 15012213

[ref46] Parniske M. (2008). Arbuscular mycorrhiza: the mother of plant root endosymbioses. Nat. Rev. Microbiol. 6, 763–775. 10.1038/nrmicro1987, PMID: 18794914

[ref47] Pozo M. J. Azcónaguilar C. (2007). Unraveling mycorrhiza-induced resistance. Curr. Opin. Plant Biol. 10, 393–398. 10.1016/j.pbi.2007.05.004, PMID: 17658291

[ref48] Qin F. Kakimoto M. Sakuma Y. Maruyama K. Osakabe Y. Tran L. S.. (2010). Regulation and functional analysis of ZmDREB2A in response to drought and heat stresses in *Zea mays* L. Plant J. 50, 54–69. 10.1111/j.1365-313X.2007.03034.x, PMID: 17346263

[ref49] Ramachandra R. A. Chaitanya K. V. Vivekanandan M. (2004). Drought-induced responses of photosynthesis and antioxidant metabolism in higher plants. J. Plant Physiol. 161, 1189–1202. 10.1016/j.jplph.2004.01.013, PMID: 15602811

[ref50] Ren A. Gao Y. Chen Y. (2004). Effects of endophyte infection on POD, SOD and PPO isozymes in perennial ryegrass (*Lolium perenne* L.) under different water conditions. Acta Ecol. Sin. 24, 1323–1329.

[ref51] Rennenberg H. Loreto F. Polle A. Brilli F. Fares S. Beniwal, and R., A. (2006). Physiological responses of forest trees to heat and drought. Plant Biol. 8, 556–571. 10.1055/s-2006-924084, PMID: 16773557

[ref52] Sannazzaro A. I. Ruiz O. A. Albertó E. O. Menéndez A. B. (2006). Alleviation of salt stress in *Lotus glaber* by Glomus intraradices. Plant Soil 285, 279–287. 10.1007/s11104-006-9015-5

[ref53] Scandalios J. G. (2005). Oxidative stress: molecular perception and transduction of signals triggering antioxidant gene defenses. Braz. J. Med. Biol. Res. 38, 995–1014. 10.1590/S0100-879X2005000700003, PMID: 16007271

[ref54] Schramm F. Larkindale J. Kiehlmann E. Ganguli A. Englich G. Vierling E.. (2006). A cascade of transcription factor DREB2A and heat stress transcription factor HsfA3 regulates the heat stress response of Arabidopsis. Plant J. 60, 759–772. 10.1111/j.1365-313X.2007.03334.x, PMID: 17999647

[ref55] Sherameti I. Venus Y. Drzewiecki C. Tripathi S. Dan V. M. Nitz I.. (2008). PYK10, a beta-glucosidase located in the endoplasmatic reticulum, is crucial for the beneficial interaction between *Arabidopsis thaliana* and the endophytic fungus *Piriformospora indica*. Plant J. 54, 428–439. 10.1111/j.1365-313X.2008.03424.x, PMID: 18248598

[ref56] Srivastava A. Guissé B. Greppin H. Strasser R. J. (1997). Regulation of antenna structure and electron transport in Photosystem II of *Pisum sativum* under elevated temperature probed by the fast polyphasic chlorophyll a fluorescence transient: OKJIP. Biochim. Biophys. Acta 1320, 95–106. 10.1016/S0005-2728(97)00017-0

[ref57] Strasser B. J. (1997). Donor side capacity of photosystem II probed by chlorophyll a fluorescence transients. Photosynth. Res. 52, 147–155. 10.1023/A:1005896029778

[ref58] Strasser R. J. Tsimillimichael M. Srivastava A. (2004). “Analysis of the chlorophyll a fluorescence transient” in Chlorophyll a Fluorescence. Vol. 19. Netherlands: Springer, 321–362.

[ref59] Tester M. Bacic A. (2005). Abiotic stress tolerance in grasses. From model plants to crop plants. Plant Physiol. 137, 791–793. 10.1104/pp.104.900138, PMID: 15761207 PMC1065378

[ref60] Timperio A. M. Egidi M. G. Zolla L. (2009). Proteomics applied on plant abiotic stresses: role of heat shock proteins (HSP). J. Proteome 71, 391–411. 10.1016/j.jprot.2008.07.005, PMID: 18718564

[ref62] Wahid A. Gelani S. Ashraf M. Foolad M. R. (2007). Heat tolerance in plants: an overview. Environ. Exp. Bot. 61, 199–223. 10.1016/j.envexpbot.2007.05.011

[ref63] Wang F. Guo Z. Li H. Wang M. Onac E. Zhou J.. (2016). Phytochrome A and B function antagonistically to regulate cold tolerance via abscisic acid-dependent jasmonate signaling. Plant Physiol. 170, 459–471. 10.1104/pp.15.01171, PMID: 26527654 PMC4704577

[ref64] Wilkins P. W. (1991). Breeding perennial ryegrass for agriculture. Euphytica 52, 201–214. 10.1007/BF00029397

[ref65] Xie Y. Han S. Li X. Amombo E. Fu J. (2017a). Ameliorates of salt stress on bermudagrass by the fungus *Aspergillus aculeatus*. Mol. Plant-Microbe Interact. 30, 245–254. 10.1094/MPMI-12-16-0263-R, PMID: 28134574

[ref66] Xie Y. Li X. Liu X. Amombo E. Chen L. Fu J. (2017b). Application of *Aspergillus aculeatus* to rice roots reduces Cd concentration in grain. Plant Soil 422, 409–422. 10.1007/s11104-017-3465-9

[ref67] Xie Y. Luo H. Du Z. Hu L. Fu J. (2014). Identification of cadmium-resistant fungi related to Cd transportation in bermudagrass [*Cynodon dactylon* (L.) Pers.]. Chemosphere 117, 786–792. 10.1016/j.chemosphere.2014.10.037, PMID: 25461949

[ref68] Xu L. Wang A. Wang J. Wei Q. Zhang W. (2017). *Piriformospora indica* confers drought tolerance on *Zea mays* L. through enhancement of antioxidant activity and expression of drought-related genes. Crop J. 5, 251–258. 10.1016/j.cj.2016.10.002

[ref69] Yamamoto Y. (2001). Quality control of photosystem II. Plant Cell Physiol. 283, 121–128. 10.1093/pcp/pce022, PMID: 11230565

[ref70] Yamamoto Y. Aminaka R. Yoshioka M. Khatoon M. Komayama K. Takenaka D.. (2008). Quality control of photosystem II: impact of light and heat stresses. Photosynth. Res. 98, 589–608. 10.1007/s11120-008-9372-4, PMID: 18937045

[ref71] Yee-Yung C. Hsiang-Chin L. Nai-Yu L. Wen-Tzu C. Chun-Neng W. Shih-Hsun C.. (2007). A heat-inducible transcription factor, HsfA2, is required for extension of acquired thermotolerance in Arabidopsis. Plant Physiol. 143, 251–262. 10.1104/pp.106.091322, PMID: 17085506 PMC1761974

[ref72] Zegaoui Z. Planchais S. Cabassa C. Djebbar R. Belbachir O. A. Carol P. (2017). Variation in relative water content, proline accumulation and stress gene expression in two cowpea landraces under drought. J. Plant Physiol. 218, 26–34. 10.1016/j.jplph.2017.07.009, PMID: 28763706

[ref73] Zhang J. H. Liu Y. P. Pan Q. H. Zhan J. C. Wang X. Q. Huang W. D. (2006). Changes in membrane-associated H^+^ -ATPase activities and amounts in young grape plants during the cross adaptation to temperature stresses. Plant Sci. 170, 768–777. 10.1016/j.plantsci.2005.11.009

[ref74] Zhu X. C. Song F. B. Liu S. Q. Liu T. D. (2011). Effects of arbuscular mycorrhizal fungus on photosynthesis and water status of maize under high temperature stress. Plant Soil 346, 189–199. 10.1007/s11104-011-0809-8

