# Supplementary material for: Exogenous Aspergillus aculeatus Enhances Drought and Heat Tolerance of Perennial Ryegrass
Source: Front Microbiol. 2021 Feb 19;12:593722. doi: 10.3389/fmicb.2021.593722 (PMC7933552; doi:10.3389/fmicb.2021.593722)
Supplement: Supplementary file 1 [file Data_Sheet_1.docx]

*HSP26.7a*

CAGTACGCTCAGATGATGCAGTACGCGGCACACATGCAGCAGGCGGCGCACGCCAGTCGGCTCCATGGCACGGCCGTCTCGGACGTGCCCAGTGATGCAGGTACGAGGAAGGGCGGAACTGCGGCGGCCGTTGGAGGACCGGGCTCGTCTCAAACAACACCTGAAACAGATAGGAAGGAGGAGTCCTGATTTATCTTCTTGTACCCGAGCAAGTCTCCACGAGTACGACTAACAAGAGTAAAACGAAACAGTAAACTGTATCTGTCAACCCCATTCTTTCGATTCGATGTCGAATAAACAGAACTCTTCCTAATTCATCAGGTTATGCAGCCATTGTCTCTGTACATTTTTCTGTTCAGTTTATTGGCTACTCGCGTCCTGAAAGAACACATCAATCACTTGGGGATGAATGTCACTTACATAAGTTAGTCGAGACGCATTAGGAGATCAAGCGACGGAACACTCACATTATTATTCCATTACTTTGCCGACTATGTACATACATCGCTACATATTCTGCGAAACGACGGCAGAGACAACAGCGCAGAAAAAGAGCACACTAGAATCTACAGAGAGCACTCACTCGCGCGGCCACAACTTGGACACTGAAACAGCTTAAATCCTCGCGGGACACAAGCTCACTGGAGCTTTCGATCACTGGACCTGCACGTCGATGACCTTGCGCTCGGTCTCCGTCTTGGGCACGGTGACGAGCAGCACGCCATTCTTGAGCTCAGCGCGCACCTGGCTCTTGTCACACTCATCCGGAAGCGCCAGGCGCATGTCGTAGGAGCTCACGCTGCGCTCCTTCCACCACCCGTCGCCCTGTCCTTCCGCGCCCTCCCCTTGGCCTTCGCTGGTTTCCTTCCTGTGCTCGCCGCGGATGACGAGTGTGTCGTCCTCCACCATCACCTTCACCTCGTCCCGCGACAGCCCGGGCATGTCGAACCGCATCTTCACCTCCTTCTCGTCCTCCATGATGTCCCAAGGCATCCGCGGCCCATCGCTCGCCGCCGCCGGCGAACGCCGTGCCGTGGGGAACCCCACCGTGTCGTCGAACAGGCGGTCCATCGTGTCCAGCATCTGCCTCATGGTTCGCATCGGCGACATCGGGTCCACCAGCCCTGCGAGGGTTAATCATGTCAGCCTCCGCTTCATCTCCGATCGTGCAACAGAGTAAGAGTTGTCGCGTGAAAGAACATCTCAGCAGCAGAGAACTTACCGAACGGGGAGATGTCGAACCCGGCGCGACGCGGGCGGCGCTGGACGGCGTTGCCCTGCTGGTTGTTGCCGCCGTTCTGGCTGACTTGCACGTCGACGGAGTTGTCCCTGTTCTCCTGCGACGCGGCGGCCACGGAGAGCGGGCGGGTTCTCCCGGCCCCGAGC

*sHsp17.8*

GTGCTGCAGATCAGCGGCGAGCGCAACAAGGAGCAGGAGGAGAAGTCCAACACCTGGCACCGCGTCGAGCGCAGCAGCGGCAAGTTTTTGCGCAGGTTCAGGCTCCCGGACAACGCCAAGACAGAGCAGATCAAGGCGTCCATGGAGAATGGCGTGCTCACGATCACCGTGCCCAAGGAGGAGGCCAAGAAGCCCGACGCCAAGCCCGTACAGATCACCGGCTAGAGACTCCGGCTTAGCTGCTCTCTGCTCACAGCCTAAATAAAATCAGCTCTTATTTGGGTGCTGCGGTGTGCGTTTGCGTGCAAGGTTGGGCTCGCTGTGTAAAACTGCATCTTTTGTCCTAGCTGATAGTTCGTTGTGCCGTAGACTGTAGGCGCTGCTTTGCGTGTGAGCCCGA

TCAGATTTCCTTTGTCTCTTTTCAAACTGTGAGCATAATGAGCTTTGTATCACGTATACTGTGCTATGTT

*DREB1A*

aaccaaaagcatccagagcttaccaagttaaaggaacagagggtactgagattgggctgatctcctagttggatttgtgaattaaatcgaagtctcaaggaagaagatgtgtcaaatcaagaaggagatgagcggggagtcgggttcgccgtgcaacggggagtactgctctccctcgacctcgtcggagcagaagcagcagacggtgtggacgaagcggcccgcggggcggacaaagttcagggagacgcggcacccggtgtaccgcggcgtgcggcgcaggggcaatgccgggcggtgggtgtgcgaggtgcgggtgccagggcggcgcgggagcaggctctgggtcggcaccttcgacactgccgagatcgccgcgcgcgcacacgacgccgccatgctcgctctcgccgccggcgacgtgtgcctcaacttcgccgactccgctgagctgctcgacatgccggcatcctcctaccgcagcctcgacgaggtgcgccacgccgtgaccgaggccgtcgaggaattcgagcggcggcaggcactgggcgaggaggacgcgctgtccggcactgagtcgtcgacgctcaccgacgacgaggagtcgtccacgccgttcgagctggacgtcctaagcgacatgggctgggacctgtactacgcgagcttggcacagggcatgctcatgtcgtctccatttctggctgcgtctgcggcgctcggggattacggcgaagccaacctcgccgatgtgccactctggagctacctgagctagttcagttgtcgccacttcaaattttacctctctccttcggtgtcgtcttggcttcggatgccaaattttggttctgtacggtcactgtttccagtttctggttatgtgatgtgatagtgcaaattggaaaaaacagagcatggatttaaaaaaaaaaaaaaaaa

peroxidase 47 (*POD47*)

GGATTACTGCAGATTGGGCTGGTCTAGCTCATGCCTCCAACATGGGCCGAAGGCTCTACAGCCCAAACTTATATGAATCAAATCATGAGGCAACACCCTCACAATAAAACTAAGAAGTTCTCAAAAAAAGAAAAACAAGAGAACTAAGAAAACGTTCGTTCAGGTTCCCCTGACAAGAAAGGTTTTGCACCCCATATCTTGGCTGCATGTGTAAGAACATATATTTTGTTTGATATATGGAGTATGTTACACGGTTGTAGTACAATCCATTTATAGAACAAGTATACATCAAATTATGCATTATACATTGTTCCTGTATATTTCACTTAATTCACCTTATTATATCCTGGACAGTGCCATGCACATTTATTGGCAAATCACCCAGCATCATCACACCTCGAGTCCTCGACAAAGTATTGTGTTTACACGCACGACAATAGATAGTTACCTTATTACCATTTCAGACGAGCTAGTTTGATTCTTGTGACGATTGATGACGCTTGTGCGCATAGTACTTAATTTACCAGCGGCCCGAGTTGACGACCCTGCACGATTTCCTGACCTCGCCATGGTCGCCCTCCTTGAGGTCGATCTGCCCCATCTTGAGCATCCCCTGCTGGAACGCGAAGAAGAAGTACCCCGGGTTCATGGCGAACATGTTCACCAGCTGCTTCGTCTCGGGCGACTCGAACAGCGTCTGGTCCGAGCTCAGAAGGCCCCTCCTCTGCTGCAGCTCCTTGAAGTAGACGCCGTCGAAGGCCGTGCTGGTCCGGTCGAACGTCGCCGTGGCCGAGTCCCCCCCGCTCTTGCACGCGGCCGCCAGCGACGAGCCCAGGCCGGAGTCCAGCGTCGCCGTCTCCGTCGTGAGCCGGGTCTTGAACGCGGCGCAGTGCGCCACGCCCAGCGTGTGCCCGCCGGAGAGCGCCACCATGTCCTGCACGTTGAAGCCGTGGGTAGCGAAGAGACTGATCAGCGACGAGGCGTTGAGGAACGGCGCCGGGAGCGCCGTGAAGGTGTCGGAGAACACCGAGCGGGACCCGTCGCGCCGCCCCGTCGGCACGCCGTAGTAGGGGCCTCCCGCGAAGAGCACCGCGTCGCGCGCCGCGAAGGCCAGCACGTCCGCGCAGGACACCACGCCCGGGCACTGCGCTTCCAGCATCTGCTTGATCTTGTCGATCACCTCGAACCCGCGCAGGCTCTTGTTCGCCGGCGCGTCCTTCTCCGCCGTGTTGCCCGCCGTCGAGTCC

superoxide dismutase (*Cu/Zn-SOD*)

CCCCTCCCCCCCAAAAAAACTTAAAAAAACTTGAATAAGTTTCTCTTGGTTATAAAGCTAGCAATAGACATTAATATCATCGCGTTCGTCCGTTTCCTTCGTCTTACACAAGAACTCTACTTGATTCTTTTCCCCCCAATATTAAAATGCCAAAATGCAAGGCCTCCTGTCCCCCCCCCCCCAAAGTATAAAAGTCAAAACTCTCCTCTCCTGTCCTCTTCTCTCTTACGTAATTAGATTAAAGCAACAGCCACCAGGCCCATAACAACCCCCGTCAAGCCAGCCTTGATACCGGCCGCACCAGCAATCGGAGCCGGAGTGTTAGAAGCCGGGCCCGTAGGAGCAGAGGTAGAAGAGCTGGTCACGGCTCCACCGGTCGGGAGAATCGTCGAGTTACCAAATCCACTCGTAGACGAAGGCTGCGTGACGCCGCCTTCCACCTTCTTGAAATCCGCGCAAGTGATTCTCGTCTTGTTGGGGTAGTGGAACACAATAGACCGGTTACCGAAAAATGCTCCAAGCCCTTCCTGAGTAGAGGCATAGAGATCCGTATAGGTCGCCTCGAAGGTGCCCTCTGCGGTGATAGGGATGGCCCCGAACTTGCCCGAGAGATCACCGACCTGGCAGGTCTCGGGGAGGGCAGCATTGCATGAGGTGGCCTCGCCGCGCTCGAAGGGATCGAGATGGGCGAGGGTCGTAGTGCAGTTACCGTTTGAAACGGGGGCAACGTGAAGGTGGTAGGGGAGAGGACCGCCCTCCTTGGGAAGGTTGCTGAGCTTGATAGTGAAGACTACGCCCTT

GCCATCCGGAGCGGCCTCGGCGCTGACGTCACCTTGGATGTTGCCGCCATTGGGGTATGCGCTCTTGAAGAAGGCTTCGGCTGGGAGCGTAGCTTTGTACTTGACGCCGACGGGGTTGGTGGAAACTATGGTGGCATTGCCGAGTTTGCCTGTCTCTGGGCCCTCCGGAGTAACAGTCGTGGTTGGTGCCGCCGAAGCTGAGGTTGAGGTCTGGGCGAAAACCTGAGTGCCGGCGACGGCGATAAAAGAGAAAGGTCGCGTCCTGCATTCCCAAGCTTGCTCGACCTCCGTTCGGTCTTTCTACGCCATCTGCTTTGAGATATCTCGACACTCTCGTTGAAATATAAAGAAATATCCATTGACAGCCATTCGTTCAACTT

iron-containing superoxide dismutase (*FeSOD*)

AAGACATGTAAATTTTTGATAATTTTTTTTCCCCTGCCCTTTTTTGTCCAGCCAGATTCATTTTCATTATTTTTCCCTATTTCAAGATCACAATACAACACAAACACACTCACTCGTACATAGTTTCTATAAATTTTCCTTTTTTGTACATACATACCATACAATCCCATCATGTAGATCCTTCCTTTTTTTGGCGGCGGCGGCCGGGGGGGATATATATGGCATTTTCCATCATATTATACAAATTGCCCTTTGGGGGTTTTTTTTTTCCTCCTCGCTCTCTCTCTGTTTTCCCAAAAGCTTCTGTCAAACACTGCGCCCCAGGAGAAAAGGGGGGCCCCTGAGTGTTTAGGACGACATCTTGGGCGCCTTGGTACCGTAAGCCAGCTGGTAGACCTTCTTCCAGTCGACCTTGTTCCACCAGTTCAACGCGAACTCGTACTTTCCGCCCTTTGCGCCCGAGCCGAACTGGTAATCCCACAGCCACACGTGCTCCCAAGTGTTCAAGCACAAAACGGGCATCAGGTCGGTACCGCCTGGCGCAAAGTTGTGTCTGCTTCTAGAGGCGTGCGATGCGTTACCGGCGCCGTAGGCAGAGTTCTCGAGGAAGCGCCTGCCAGCCGCGAGACCCTGGGGCGTG

CTTGTGCCGACGTCGGTGTTGGTGTCGATCTCCTGGCGACGCCAGTGGGCGCCCGGGTAAGGGGAGCCGGCGAGGTAAGTGGTGAGGACCTTGTAAGCGTTTGGAGCGCCGGGGAGGTCCGTCTTGACGAGCCAGACGAAGCCCGGGCCGAACATGGCCGAGGCGGTCTTGACCATCTCGGCTTTGAGTGTCTCGATCGAGCCGAAGGACCGGATCAGGTCGGCCTGGAGATCTTCGGGCATGGCCACCTCGCCCGCTGGGGAGATGCCGTTGAAGAAGAAGTGGTTGTTGTGCGCCATGCTGGCGTAGTTGAAAATGGAGGCATATTGGGGTTCGCGGGCGGTGGCGAGAACAATGTTCTTGAGGTCTTTGGTCTCGAAGTCGGTTTCGGCCGTCAAGC

CGTTTAGTCTTTGCAGGACGTGGGTCTGGTAGGCATACCAAGCGATGGTCAAGCCGTCGGGGGAGAGAAGCCCGGGAATTCCTTTTTGTTGATCGAAGTTCTCGAGGGCAGGGATTGTATGCTGTGATCTTATTTGTTGGGCGAGCGGGAGGAGCTTGGAGGAGGCTTGGGTGGGCGCAATGAGGGATGGCCTCAGCGATATTCTTAACCTTGGGCGGAACATTGTGTCTGGTGGTTATGGTGTTCAATTGGCGAATGTGCCCGCCTCGTCGTCGTCGTCGTCGTCGATTCTACCAATATGCGA
